# A New Family of HEAT-Like Repeat Proteins Lacking a Critical Substrate Recognition Motif Present in Related DNA Glycosylases

**DOI:** 10.1371/journal.pone.0127733

**Published:** 2015-05-15

**Authors:** Elwood A. Mullins, Rongxin Shi, Lyle A. Kotsch, Brandt F. Eichman

**Affiliations:** Department of Biological Sciences and Center for Structural Biology, Vanderbilt University, Nashville, Tennessee, United States of America; University of Massachusetts Medical School, UNITED STATES

## Abstract

DNA glycosylases are important repair enzymes that eliminate a diverse array of aberrant nucleobases from the genomes of all organisms. Individual bacterial species often contain multiple paralogs of a particular glycosylase, yet the molecular and functional distinctions between these paralogs are not well understood. The recently discovered HEAT-like repeat (HLR) DNA glycosylases are distributed across all domains of life and are distinct in their specificity for cationic alkylpurines and mechanism of damage recognition. Here, we describe a number of phylogenetically diverse bacterial species with two orthologs of the HLR DNA glycosylase AlkD. One ortholog, which we designate AlkD2, is substantially less conserved. The crystal structure of *Streptococcus mutans* AlkD2 is remarkably similar to AlkD but lacks the only helix present in AlkD that penetrates the DNA minor groove. We show that AlkD2 possesses only weak DNA binding affinity and lacks alkylpurine excision activity. Mutational analysis of residues along this DNA binding helix in AlkD substantially reduced binding affinity for damaged DNA, for the first time revealing the importance of this structural motif for damage recognition by HLR glycosylases.

## Introduction

DNA is chemically modified by agents of both exogenous and endogenous origins to produce oxidized, alkylated, and deaminated nucleobases. If left unrepaired, aberrant nucleobases can interfere with DNA replication and transcription, potentially causing mutations and cell death [1]. Many of these modified bases are excised by lesion-specific DNA glycosylases that cleave the *N*-glycosidic bond linking the damaged base to the phosphoribose backbone [2,3]. The resulting apurinic/apyrimidinic (AP) sites are subsequently processed by AP endonuclease, DNA polymerase, and DNA ligase activities in the base excision repair (BER) pathway to restore undamaged DNA [4–6].

DNA glycosylases are faced with the challenge of discriminating their target lesions from an excess of undamaged DNA. All glycosylases are capable of anchoring non-specifically to the DNA phosphoribose backbone, presumably as a means of sliding along the duplex in search of their targets [7]. In addition to this non-specific binding, glycosylases have evolved a common mechanism to recognize damaged nucleobases, whereby a DNA penetrating element probes the duplex to exploit structural and/or energetic differences between normal and aberrant base pairs [8–12]. These differences allow the enzyme to capture a specific DNA conformation and form additional contacts that provide a secondary method of modified base recognition. This ultimate substrate recognition step typically involves the damaged nucleotide being flipped from the duplex and into an active site binding pocket.

The HEAT-like repeat (HLR) DNA glycosylases exist in all domains of life and are structurally and mechanistically distinct from their base-flipping counterparts [13–16]. These enzymes are composed of tandem helical HLR units that form an overall solenoid shape with a concave, positively charged DNA binding surface, and represent the first example of such an architecture to support DNA binding or catalytic activity. The first HLR glycosylases to be identified, *Bacillus cereus* AlkC and AlkD, are specific for positively charged alkylpurine lesions [17]. Although the molecular basis for this specificity is still not well understood, AlkD has been shown to recognize aberrant DNA by a mechanism that does not involve flipping the modified base into an active site pocket on the enzyme [14,15]. Instead, the extensive contacts between the concave surface and the DNA backbone presumably provide the energy necessary to sense non-Watson-Crick base pairs [18]. The importance of interactions with the DNA backbone is underscored by the fact that AlkD makes only one contact with a nucleobase. An N-terminal α-helix (the “B-helix”) penetrates the minor groove to form a hydrogen bond with the base pair adjacent to the lesion. However, the significance of this B-helix interaction to damage specificity has not been established.

Recently, the HLR superfamily was expanded by inclusion of *B*. *cereus* AlkF and AlkG. These orthologs lack base excision activity despite a high degree of structural similarity to AlkD [19]. Instead, AlkF and AlkG possess the distinct ability to bind branched DNA structures, which has been attributed to a β-hairpin motif unique to the AlkF and AlkG families [19]. Seemingly, the HLR architecture has been adapted for a number of diverse nucleic acid substrates. Although the rationale for such diversity and the associated cellular roles remain unclear, characterization of the various protein families provides an opportunity to understand the structural mechanisms of DNA damage recognition by this unique protein architecture.

Here, we describe a new family of proteins related to the HLR DNA glycosylases that lacks DNA binding specificity as well as alkylpurine excision activity. AlkD2 was identified in a number of phylogenetically diverse bacterial species, frequently accompanied by a second, more conserved ortholog of AlkD. Crystallographic analysis revealed that *Streptococcus mutans* AlkD2 is structurally similar to AlkD but lacks the DNA penetrating B-helix. Mutational analysis of AlkD using electrophoretic mobility shift assays revealed for the first time that the B-helix is not only important but critical for DNA damage recognition. This work establishes that the unique N-terminal helical bundle in the HLR enzymes is a crucial factor in defining substrate preference, and provides further evidence that the HLR scaffold has evolved multiple distinct functions.

## Materials and Methods

### Evolutionary analysis

Orthologs of *Streptococcus mutans* (Sm)AlkD and SmAlkD2 were identified using a BLAST [20] search of all non-redundant bacterial proteins in the NCBI database (S1 Table). Representative sequences were selected from diverse species and aligned using MUSCLE [21,22]. The alignment was manually adjusted before a neighbor-joining phylogenetic tree was computed, incorporating Poisson correction to calculate evolutionary distances, in MEGA6 [23]. The robustness of the tree was assessed by 1,000 bootstrap replications with ambiguous positions removed from each sequence pair, leaving 265 positions in the final dataset.

### Protein purification

The SmAlkD2 gene was amplified from *S*. *mutans* Clarke (ATCC 25175) genomic DNA and ligated into a modified pET27 expression vector (Novagen) encoding a Rhinovirus 3C (PreScission protease) cleavable hexahistidine tag. Recombinant protein was overproduced in *Escherichia coli* Rosetta 2 cells at 16°C upon addition of 0.4 mM IPTG. Cells were harvested from LB medium by centrifugation, resuspended in Lysis Buffer (50 mM Tris∙HCl pH 8.5, 500 mM NaCl, and 10% (v/v) glycerol), and lysed at 20,000 psi with an Emulsifier C3 homogenizer (Avestin). Cleared lysate was applied to a Ni-NTA column (Qiagen) equilibrated in Lysis Buffer. The column was then washed and eluted with Lysis Buffer containing 20 mM and 500 mM imidazole, respectively. Pooled fractions were supplemented with 2 mM DTT and 0.1 mM EDTA prior to overnight cleavage of the hexahistidine tag. Cleaved protein was then diluted 10-fold in Buffer A (50 mM Tris∙HCl pH 8.5, 10% (v/v) glycerol, 2 mM DTT, and 0.1 mM EDTA) and loaded onto a heparin Sepharose column (GE Healthcare) equilibrated in Buffer A. The column was washed with Buffer A containing 50 mM NaCl and eluted by a linear increase to Buffer A containing 1 M NaCl. Pure SmAlkD2 was passed through a Superdex 200 column (Pharmacia) equilibrated in 20 mM Tris∙HCl pH 8.5, 150 mM NaCl, 10% (v/v) glycerol, 2 mM DTT, and 0.1 mM EDTA; concentrated to 10 mg/mL by ultrafiltration; and flash-frozen in liquid nitrogen before being stored at −80°C.

*Bacillus cereus* (Bc)AlkD was purified as previously described [14]. BcAlkD mutants were generated using the Q5 mutagenesis kit (New England Biolabs), overproduced at 16°C upon addition of 0.4 mM IPTG, and purified in the same manner as wild-type BcAlkD (S1 Fig).

### Thermal melting

Structural integrity of protein constructs was verified by monitoring changes in molar ellipticity at 222 nm as mixtures containing 7.5 μM protein, 50 mM HEPES pH 7.5, 100 mM KCl, and 10% (v/v) glycerol were heated at 1°C/min. Melting temperatures (*T*_m_) were determined from second-order derivatives of polynomial functions fit to the data (S2 Table).

### Protein crystallization, X-ray data collection, and structure determination

SmAlkD2 crystals were grown using the hanging-drop vapor-diffusion method. SmAlkD2 was incubated at 4°C for 30 min with an oligodeoxynucleotide duplex [d(TGTCCA(THF)GTCT)/d(AGACTTGGACA)] containing a tetrahydrofuran (THF) abasic site mimetic. Crystallization drops were prepared from 1 μL of protein/DNA solution [290 μM SmAlkD2 and 350 μM oligodeoxynucleotide duplex] and 1 μL of reservoir solution [18% (w/v) PEG 3350 and 200 mM sodium phosphate pH 4.7]. Drops were equilibrated at 21°C against 500 μL of reservoir solution. Crystals appeared within 1 day and grew to full size in 2 to 4 days. Crystals were harvested and flash-cooled in liquid nitrogen after 5 days.

X-ray diffraction data were collected at beamline 21-ID-F at the Advanced Photon Source (Argonne National Laboratory) and processed using HKL2000 [24]. Data collection statistics are provided in S3 Table. Phases were determined by molecular replacement using Phaser [25] to unambiguously position an incomplete structure of SmAlkD2 (PDB: 3L9T) from *S*. *mutans* UA159. Simulated annealing in PHENIX [26] substantially improved the σ_A_-weighted 2m*F*_o_−D*F*_c_ and m*F*_o_−D*F*_c_ maps, revealing electron density for residues 0–206 (C-terminus), including a non-native N-terminal residue from the cleaved tag (residue 0) and the loop between helices A and C (residues 15–19) that were not present in the search model. Further improvement was made by manual placement of atoms in Coot [27] and refinement of atomic coordinates and temperature factors in PHENIX. The final SmAlkD2 model was validated using MolProbity [28] and contained no residues in disallowed regions of the Ramachandran plot. Refinement and validation statistics are given in S3 Table. Atomic coordinates and structure factors were deposited in the Protein Data Bank (PDB: 4X8Q).

### DNA binding

Binding of a ^32^P-labelled oligodeoxynucleotide duplex [d(GACCACTACACC(G/THF)ATTCCTTACAAC)/d(GTTGTAAGGAAT(C/T)GGTGTAGTGGTC)] containing a centrally located G•C base pair, G•T mismatch, or THF•C abasic site was measured using electrophoretic mobility shift assays (EMSA). Protein (0–50 μM) was equilibrated with 100 pM ^32^P-DNA, 20 mM Tris•HCl pH 7.5, 100 mM NaCl, 5% (v/v) glycerol, 2 mM DTT, 0.1 mM EDTA, and 0.1 mg/mL BSA at 20°C for 30 min. Free and bound DNA were separated on a native polyacrylamide gel (5% acrylamide, 45 mM Tris, 45 mM boric acid, and 1 mM EDTA) run at 200 V and 20°C for 1 h. Equilibrium dissociation constants (*K*_d_) were determined by fitting the data to standard one-site (Eq 1) or two-site (Eq 2) binding models, where *B* is fractional occupancy of the receptor, *n* is fractional capacity of the receptor site, and L is free ligand.

$$B=\frac{n_{1}}{1+\frac{K_{\text{d}1}}{\left[\text{L}\right]}}$$ (1)

$$B=\frac{n_{1}}{1+\frac{K_{\text{d}1}}{\left[\text{L}\right]}}+\frac{n_{2}}{1+\frac{K_{\text{d}2}}{\left[\text{L}\right]}}$$ (2)

### Base excision

Excision of *N*3-methyladenine (3mA) and *N*7-methylguanine (7mG) from methylated calf thymus DNA was quantitated by HPLC-MS/MS as previously described [29]. Reactions containing 5 μM enzyme, 10 μg DNA, 50 mM HEPES pH 7.5, 100 mM KCl, 10 mM DTT, 2 mM EDTA, and 0.1 mg/mL BSA were performed at 37°C for 1 h.

## Results

### AlkD2 is present in phylogenetically diverse bacteria

**Fig 1:**
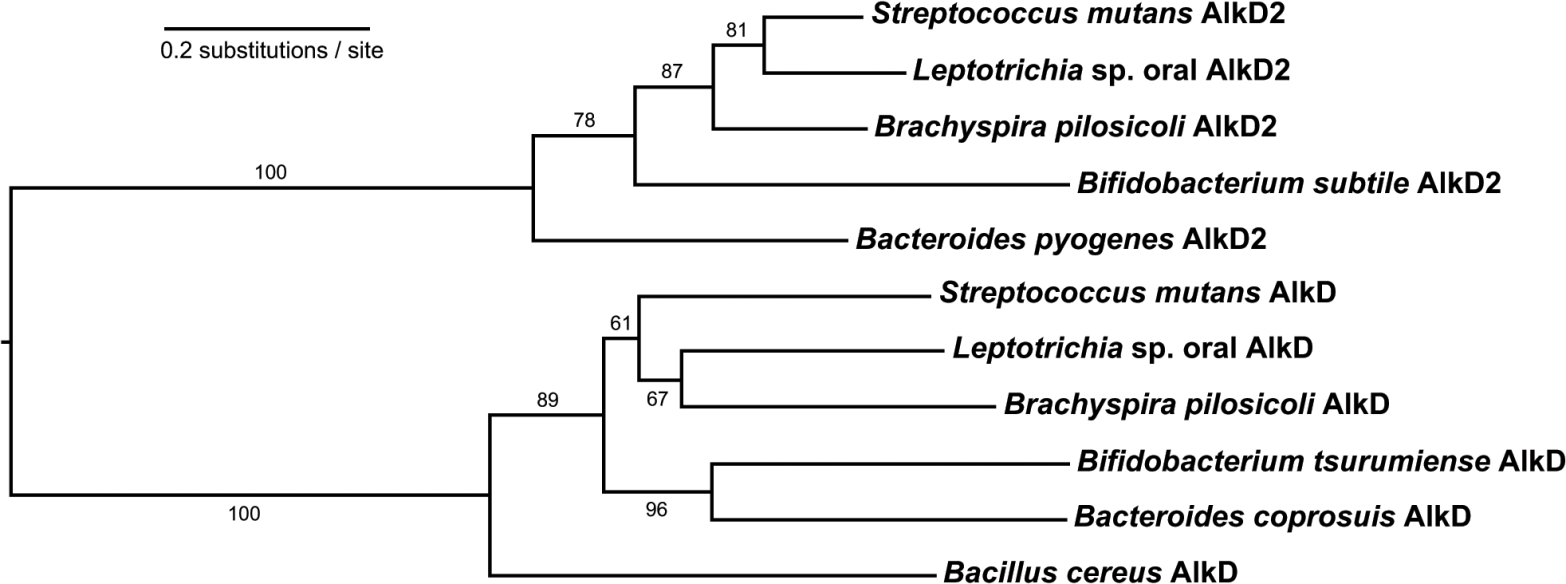
Phylogenetic history of AlkD and AlkD2 across diverse bacterial phyla. Branch lengths represent rates of protein evolution in units of amino acid substitutions per site. Numbers above the branches indicate the degree of bootstrap support in 1,000 replicates.

**Fig 2:**
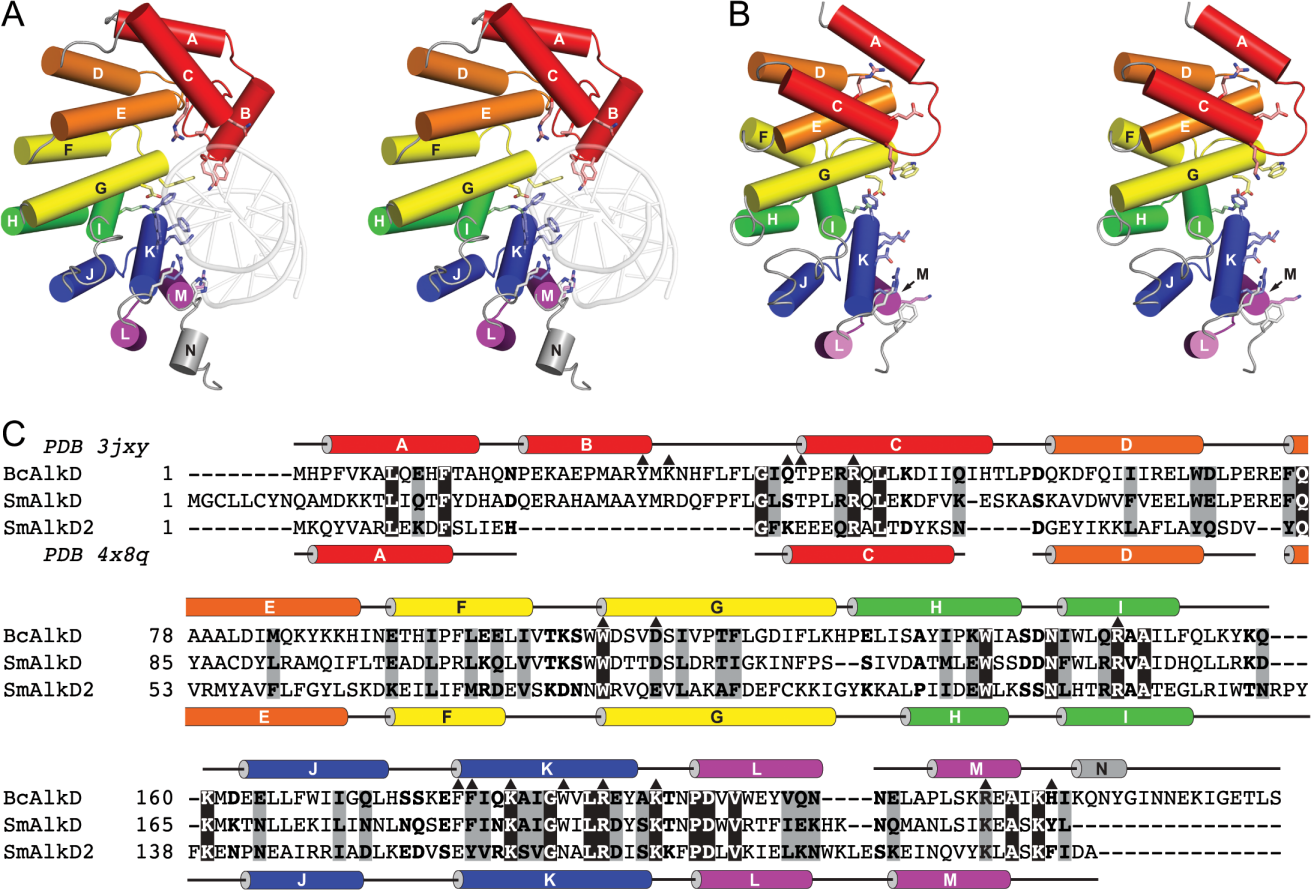
Comparison of BcAlkD and SmAlkD2. (A,B) Stereodiagrams of crystal structures of BcAlkD bound to DNA containing a G•T mismatch (PDB: 3JXY, panel A) and SmAlkD2 (PDB: 4X8Q, panel B). DNA binding residues in BcAlkD and corresponding residues in SmAlkD2 are shown as sticks. Proteins are colored by HLR unit. (C) Structure-based sequence alignment of BcAlkD, SmAlkD, and SmAlkD2. Secondary structural elements are indicated for BcAlkD and SmAlkD2. DNA binding residues shown in panel A are indicated by black triangles.

All four previously known HLR families—AlkC, AlkD, AlkF, and AlkG—were discovered in *B*. *cereus* [17,19]. We identified a fifth family, which we designate AlkD2, not present in *B*. *cereus* but spread across phylogenetically diverse bacteria, commonly in species which also possess AlkD (S1 Table). AlkD2 shares less than 20% sequence identity with BcAlkD, suggesting that this family could play a distinct role in cellular metabolism. Evolutionary analysis suggested that AlkD and AlkD2 diverged from a distant common ancestor following a gene duplication (Fig 1). Alignment of AlkD and AlkD2 sequences revealed a modest but significant degree of similarity between the two families, primarily in the C-terminal halves of the proteins. The most notable difference is a deletion in the AlkD2 family that corresponds to the B-helix in AlkD (Fig 2C and S2 Fig).

### AlkD2 lacks the B-helix of AlkD

In an effort to understand the molecular differences between the AlkD and AlkD2 families, we determined the crystal structure of AlkD2 using a previously determined but incomplete structure of SmAlkD2 (PDB: 3L9T) obtained by another group. This structure lacked atomic coordinates for residues 15–19, which belong to a loop that corresponds to the B-helix in AlkD (Fig 2C and S2 Fig). We developed improved crystallization conditions that provided electron density for the missing parts of the search model, permitting a more complete and detailed structural analysis (S3 Fig). Both BcAlkD and SmAlkD2 are primarily composed of similar tandem helical HLR units. However, the non-HEAT helical motifs at their N-termini substantially differ. In BcAlkD, this motif is made of three helices (ABC), two of which, helices B and C, interact with DNA (Fig 2A). In SmAlkD2, the N-terminal capping motif lacks helix B, and helices A and C are instead connected by a short loop (Fig 2B and S3 Fig). The loss of helix B is accompanied by a ~10 Å translation of helix C toward helix E. The positions of the corresponding residues in the sequence alignment suggest that helix C is also rotated by 180° about its longitudinal axis. These spatial differences would eliminate or alter interactions between helix C of BcAlkD and DNA. DNA binding interactions outside the N-terminal capping motif are more conserved. Five of eleven DNA binding residues located throughout the HLR units are identical, while three more are similar and likely functionally equivalent (Fig 2C). Thus, at a structural level, the main difference between AlkD and AlkD2 is the presence of the B-helix in AlkD, which provides the only nucleobase contact and helps orient the N-terminal helical cap for additional interactions with the DNA backbone.

### AlkD2 is not an alkylpurine DNA glycosylase

**Fig 3:**
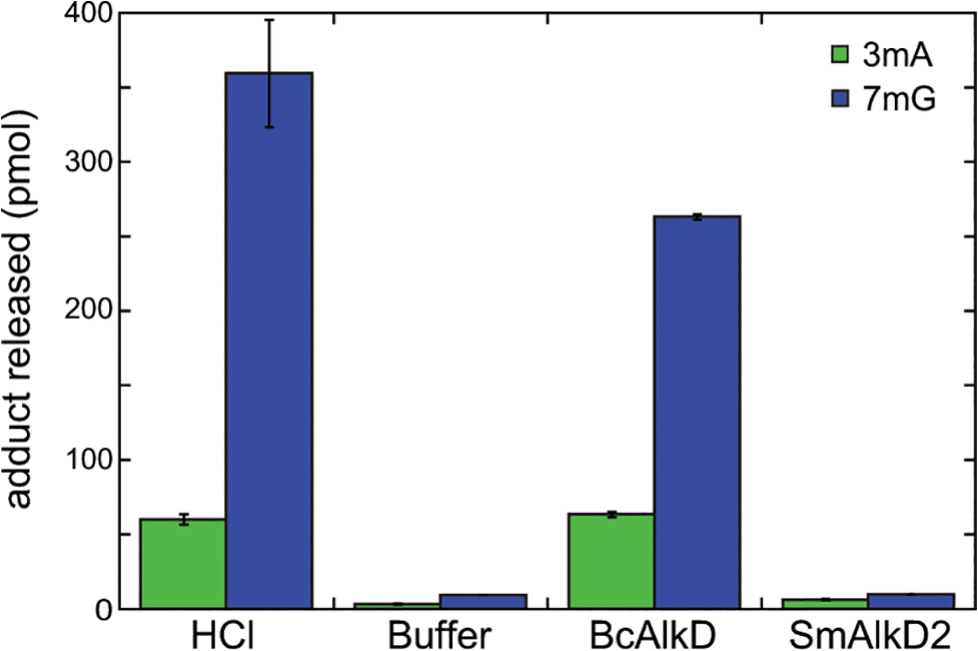
Excision of methylpurine adducts from genomic DNA. HCl and buffer controls indicate the upper and lower limits for removal of 3mA (green) and 7mG (blue) by BcAlkD and SmAlkD2. Error bars indicate the standard deviations from the mean from three independent measurements.

Given the evolutionary divergence between AlkD and AlkD2 and the lack of the DNA-binding B-helix, we examined the ability of SmAlkD2 to excise 3mA and 7mG from methylated genomic DNA. Under our experimental conditions, BcAlkD removed all 3mA and most 7mG relative to HCl-catalyzed depurination (Fig 3). Conversely, SmAlkD2 failed to increase the amounts of 3mA and 7mG relative to a buffer-only control. Thus, AlkD2 does not appear to support alkylpurine excision activity, at least not against the same cationic substrates removed by AlkD. Neither enzyme excised *N*1-methyladenine or *O* ^6^-methylguanine (data not shown), consistent with the previously reported specificity of BcAlkD for positively charged *N*3- and *N*7-methylpurine lesions [17,29].

### AlkD2 has greatly reduced affinity for aberrant DNA

**Fig 4:**
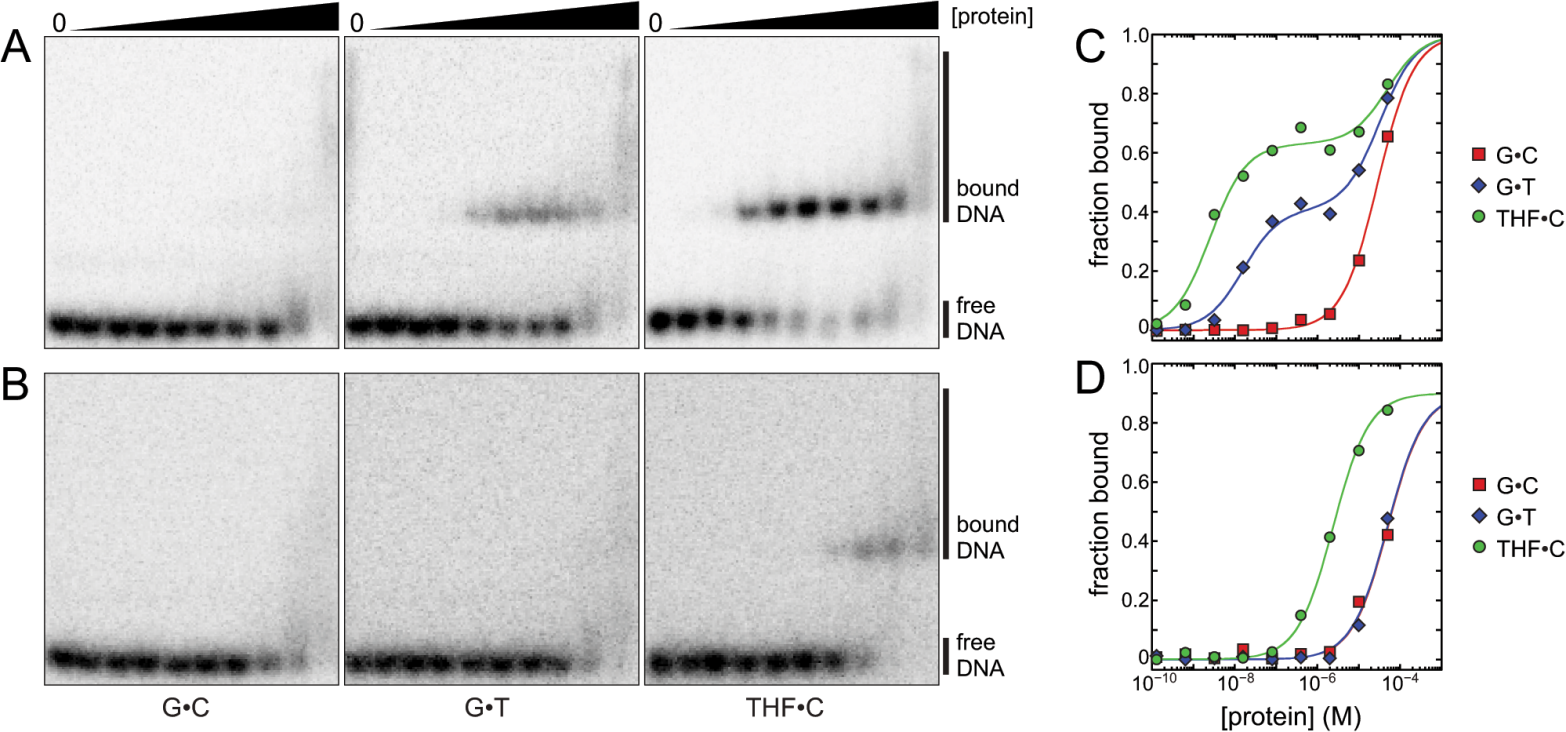
DNA binding by BcAlkD and SmAlkD2. (A,B) Representative native gels showing binding of 25-mer dsDNA containing a central G•C base pair, G•T mismatch, or THF•C abasic site by BcAlkD (panel A) or SmAlkD2 (panel B). Binding reactions contained 0, 0.13, 0.64, 3.2, 16, 80, 400, 2,000, 10,000, or 50,000 nM protein and 100 pM DNA. (C,D) Quantitation of DNA binding in panels A and B. Red squares, G•C-DNA; blue diamonds, G•T-DNA; green circles, THF•C-DNA. Experiments were performed in triplicate.

To ascertain whether loss of alkylpurine excision activity by SmAlkD2 was the result of an inability to bind DNA, we measured binding affinity for oligodeoxynucleotide duplexes containing a centrally located G•C base pair, G•T mismatch, or THF•C abasic site. We had previously determined using fluorescence anisotropy that BcAlkD binds fluorescein-labeled substrates with dissociation constants in the low-micromolar range, with only a 2-fold difference between unmodified and modified DNA. Using an EMSA, we found that both BcAlkD and SmAlkD2 bound unmodified DNA with the same weak low-micromolar affinity determined by the fluorescence anisotropy assay (Fig 4 and Table 1). Unlike the fluorescence anisotropy assay, however, the EMSA revealed that BcAlkD recognizes G•T-DNA and THF•C-DNA with 1,700-fold and 13,000-fold higher affinity than G•C-DNA, exhibiting dissociation constants in the low-nanomolar range (Fig 4A and 4C and Table 1). Specific and non-specific binding produced sharp bands and broad smears, respectively, which correlated with two distinct binding transitions for mismatched and abasic DNA. The second, weaker binding transition is of comparable affinity to that of the single transition observed with unmodified DNA. In contrast, SmAlkD2 bound G•T-DNA with the same low-micromolar affinity as G•C-DNA and bound THF•C-DNA only 20-fold more tightly (Fig 4B and 4D and Table 1). This small preference for abasic DNA, while clearly indicative of weak specific recognition, is insufficient to produce two distinct binding transitions.

**Table 1:** DNA binding affinities.

	G•C-DNA		G•T-DNA		THF•C-DNA	
	*K*_d1_ (M)	*K*_d2_ (M)	*K*_d1_ (M)	*K*_d2_ (M)	*K*_d1_ (M)	*K*_d2_ (M)
**BcAlkD** ^**a**^	(1.9 ± 1.2) × 10^−5^	—	(1.1 ± 0.5) × 10^−8^	(3.0 ± 1.0) × 10^−5^	(1.5 ± 1.1) × 10^−9^	(3.4 ± 1.5) × 10^−5^
**SmAlkD2**	(6.7 ± 2.5) × 10^−5^	—	(5.2 ± 0.6) × 10^−5^	—	(3.4 ± 1.1) × 10^−6^	—

^a^ Data were fit to a one- or two-site binding model. Values represent the averages and standard deviations from the mean from three experiments.

**Fig 5:**
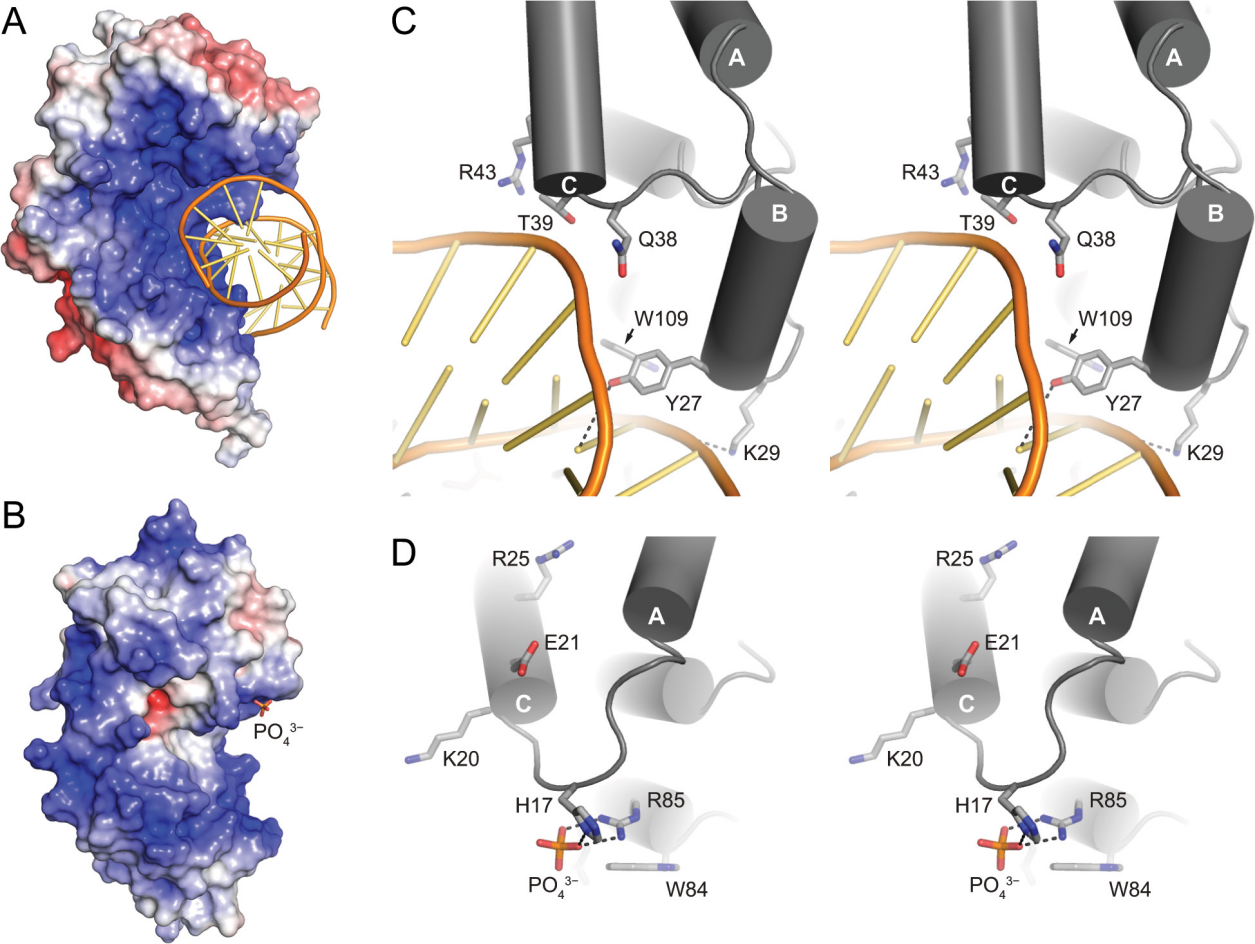
BcAlkD and SmAlkD2 binding interactions. (A,B) Solvent-accessible surfaces colored by electrostatic potential of BcAlkD bound to DNA containing a G•T mismatch (PDB: 3JXY, panel A) and SmAlkD2 bound to phosphate (PDB: 4X8Q, panel B). The saturation of the colors (red, negative; blue, positive) is proportional to the degree of electrostatic charge from −7 to +7 *k*_B_ *T*/*e*_C_. Electrostatic surfaces were calculated using PDB2PQR [30,31] and APBS [32]. (C,D) Stereodiagrams of the interactions between the ABC motif of BcAlkD and DNA (panel C) and the A−C motif of SmAlkD2 and phosphate (panel D). Hydrogen-bonding interactions are indicated with dotted lines.

While BcAlkD and SmAlkD2 are remarkably structurally similar, local differences likely account for the greatly reduced specific recognition of modified DNA by SmAlkD2. Of the five DNA binding residues located in the ABC motif of BcAlkD (Fig 5C), only one is appropriately positioned to interact with DNA in the A−C motif of SmAlkD2 (Fig 5D). The position of a bound phosphate ion in the SmAlkD2 structure, however, suggests that SmAlkD2 may interact with DNA through two residues, His17 and Arg85, not shared with BcAlkD (Fig 5D and S3 Fig). His17 is located on the loop connecting helices A and C, and Arg85 is positioned near the N-terminus of helix G. Of these two residues, only Arg85 is invariant in the AlkD2 family.

Electrostatic interactions are a universal feature of non-specific protein-DNA interactions. Correspondingly, BcAlkD has a highly positively charged, concave binding surface (Fig 5A) that is distinct from that of HEAT-repeat proteins, which have a similar structural architecture but no affinity for DNA [15,16]. Interestingly, SmAlkD2 retains a largely positively charged concave surface (Fig 5B) despite its low sequence similarity to AlkD. We speculate that this shared electrostatic feature is responsible for the similar non-specific, low-micromolar affinity of BcAlkD and SmAlkD2 for unmodified DNA.

### The B-helix enhances recognition of DNA damage

**Fig 6:**
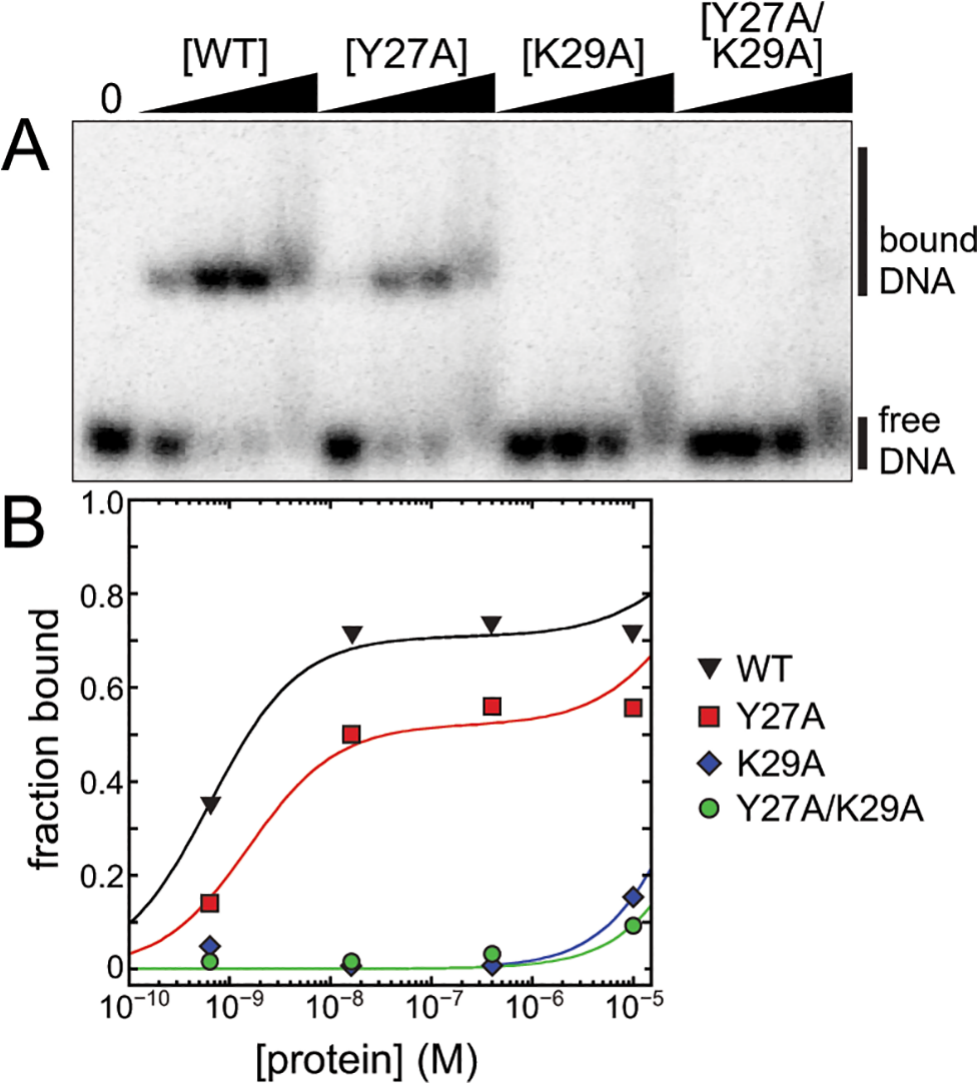
Binding of aberrant DNA by wild-type and mutant BcAlkD. (A) Native gel showing binding of wild-type and B-helix mutants of BcAlkD to 25-mer dsDNA containing a central THF•C abasic site. Binding reactions contained 0, 0.64, 16, 400, or 10,000 nM protein and 100 pM DNA. (B) Quantitation of DNA binding in panel A. Black triangles, BcAlkD-WT; red squares, BcAlkD-Y27A; blue diamonds, BcAlkD-K29A; green circles, BcAlkD-Y27A/K29A.

The lack of a B-helix in SmAlkD2 and an inability to bind modified DNA implicate this structural element in damage recognition. Indeed, Tyr27 on the B-helix provides the only nucleobase contact in BcAlkD. It was therefore somewhat surprising that we previously found using the fluorescence anisotropy assay that substitution of Tyr27 with phenylalanine or alanine did not significantly affect DNA binding or base excision by BcAlkD [18]. In light of the new observation of specific DNA binding using the EMSA, we repeated the BcAlkD mutational analysis, this time also testing Lys29, which forms a salt bridge with the DNA backbone adjacent to the Tyr27-nucleobase contact (Fig 5C). Consistent with our previous analysis [18], the Y27A mutant showed a modest 2–5-fold reduction in specific binding affinity relative to wild-type BcAlkD (Fig 6). However, substitution of Lys29 with alanine, either alone or together with Y27A, abolished specific recognition of abasic DNA, reducing affinity to approximately that for normal DNA. These data confirm the importance of the B-helix to lesion recognition in the AlkD family of enzymes and indicate that Lys29 is the primary contributor to DNA binding by the B-helix in BcAlkD. Lysine and arginine are most often found in this position in AlkD homologs (S2 Fig), suggesting that the affinity of the B-helix for DNA is electrostatic in nature. In support of this, the one AlkD homolog in S2 Fig. without a basic side chain at this position (leucine in *Leptotrichia* sp. oral) instead has a lysine in a different position along the B-helix that appears to be appropriately positioned to form a salt bridge with the DNA backbone (S4 Fig).

## Discussion

Here, we describe a new protein family related to the HLR DNA glycosylase AlkD and examine structural requirements for damage recognition by the HLR architecture. AlkD is broadly distributed throughout all domains of life, and related proteins lacking glycosylase activity are present in select bacteria [15,19]. We identified numerous, diverse bacterial species that possess two orthologs of AlkD and showed through phylogenetic analysis that the AlkD and AlkD2 families diverged from a distant common ancestor. Structural characterization revealed that AlkD2 lacks the DNA binding B-helix present in AlkD, and biochemical and mutational studies linked this missing structural element to loss of aberrant DNA binding affinity.

The EMSA binding data revealed for the first time substantial differences in the binding affinity of BcAlkD for normal and non-Watson-Crick DNA. Our previous quantitation of BcAlkD-DNA binding by fluorescence anisotropy indicated low-micromolar affinity for both unmodified and modified DNA [14,18]. This was surprising since we were only able to crystallize AlkD with DNA containing a lesion or a mismatch [15]. The much stronger low-nanomolar binding affinities determined by EMSA for mismatched and abasic DNA are consistent with our previous crystallographic data and, more importantly, show that the HLR architecture is able to distinguish normal nucleobases from lesions, almost entirely through interactions with the DNA backbone. We speculate that convolution of tight specific binding and loose non-specific binding by fluorescence anisotropy resulted in an averaged, intermediate apparent affinity.

The ability to measure lesion specific binding also provided a means to quantify loss of binding specificity upon mutation of BcAlkD, as significant mutational effects were also obscured in the fluorescence anisotropy assay [14,18]. Mutation of the two binding residues on the B-helix resulted in unequal losses of binding affinity. Even though Tyr27 is the only residue to directly contact a nucleobase, the BcAlkD-Y27A mutant bound abasic DNA with less than a 10-fold loss of affinity. Conversely, the BcAlkD-K29A mutant, which disrupts a salt bridge with a DNA phosphate, bound abasic DNA at least 10,000 times more weakly than wild-type BcAlkD. These results are again consistent with a binding model in which interactions with the DNA backbone are the primary determinants of damage recognition. These backbone interactions are not strictly electrostatic in nature since the charge potentials of BcAlkD and SmAlkD2 concave surfaces are not substantially different. Furthermore, in addition to contacts from the N-terminal non-HEAT helical motif, AlkD2 lacks three DNA binding contacts distributed throughout the HLR units. The positions of BcAlkD DNA binding residues Phe179, Tyr187, and His220 are occupied by SmAlkD2 residues Glu158, Asn166, and Phe203, respectively. Thus, any or all of these altered binding residues may be responsible for the reduced DNA binding affinity and lack of alkylpurine excision activity of AlkD2. Of primary importance is BcAlkD Tyr187, which is invariant in the AlkD family and likely plays an important role in substrate recognition and/or catalysis.

The diversity of bacterial species that possess both AlkD and AlkD2 suggests that both proteins were present before bacteria began extensive speciation over 2.5 billion years ago, and that both proteins have been retained in these species for that considerable duration [33,34]. This would seem unlikely if AlkD and AlkD2 had not evolved unique biological functions. The shared weak non-specific affinity of both AlkD and AlkD2 may indicate that AlkD2 recognizes a yet-to-be-determined DNA substrate. For example, AlkF and AlkG, which also lack glycosylase activity, have been shown to preferentially bind branched DNA structures using a β-hairpin motif that is absent in AlkD and AlkD2, although the biological function associated with this DNA specificity is unknown [19]. Alternatively, weak DNA binding could be a vestige that remained after AlkD2 acquired a role unrelated to DNA binding, or itself could be a requirement for an unknown function. Related to this, *Schizosaccharomyces pombe* and related fission yeast possess two paralogs of the alkylpurine DNA glycosylase Mag. Only Mag1 supports base excision activity while the non-catalytic Mag2 weakly and transiently binds DNA [35–37], which has been postulated to allow Mag2 to protect cytotoxic AP sites [37]. AlkD2, which also exhibits weak specific affinity for abasic DNA, may have a similar function. Although additional studies will be required to establish the role of AlkD2, this work further expands the repertoire of HLR proteins beyond alkylpurine excision.

## Supporting Information

S1 Fig Determination of protein purity by SDS-PAGE. (PDF)

S2 Fig Alignment of the ABC and A−C motifs of phylogenetically diverse orthologs of SmAlkD and SmAlkD2. (PDF)

S3 Fig Electron density for SmAlkD2. (A) Complete protein. (B) A−C motif. The 1.7-Å annealed composite omit map was calculated from the final SmAlkD2 model and contoured at 1σ.
(PDF)

S4 Fig Alternative DNA binding contacts on the B-helix. (A) *B*. *cereus* AlkD. (B) *S*. *mutans* AlkD. (C) *L*. sp. oral AlkD. Homology models in panels B and C were generated from an X-ray crystal structure of BcAlkD (PDB: 3JXY) using SWISS-MODEL [38]. Hydrogen-bonding interactions are indicated with dotted lines.
(PDF)

S1 Table Representative AlkD2 orthologs. (PDF)

S2 Table Protein melting temperatures. (PDF)

S3 Table X-ray data collection and refinement statistics. (PDF)
