# Supplementary figures and images for: A New Family of HEAT-Like Repeat Proteins Lacking a Critical Substrate Recognition Motif Present in Related DNA Glycosylases
Source: PLoS One. 2015 May 15;10(5):e0127733. doi: 10.1371/journal.pone.0127733 (PMC4433238; doi:10.1371/journal.pone.0127733)

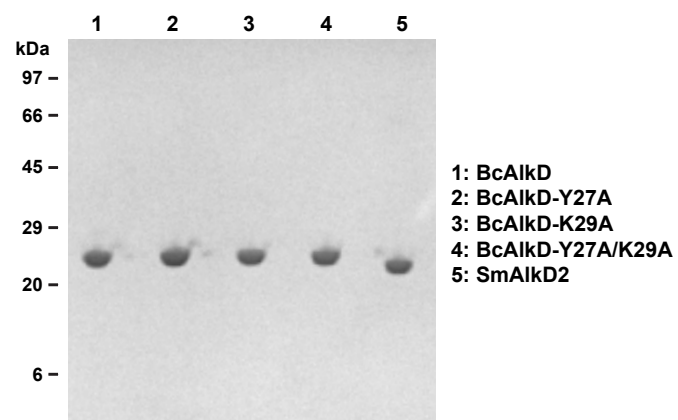

**Figure S1. Determination of protein purity by SDS-PAGE.**

Supplement: S1 Fig — (PDF) [file pone.0127733.s001.pdf]
