# Supplementary material for: A New Family of HEAT-Like Repeat Proteins Lacking a Critical Substrate Recognition Motif Present in Related DNA Glycosylases
Source: PLoS One. 2015 May 15;10(5):e0127733. doi: 10.1371/journal.pone.0127733 (PMC4433238; doi:10.1371/journal.pone.0127733)
Supplement: S2 Fig — (PDF) [file pone.0127733.s002.pdf]

```
                                       Helix A                                    Helix C
S. mutans AlkD2        ----------MKQYV---ARLEKDFSLIEH------------------GFKEEEQRALTDYKSN-----
L. sp. oral AlkD2      ----------MKEYI---TSLEKEFSLIEN------------------GFKEEEKKALADYKSN-----
B. pilosicoli AlkD2    ----------MKDYI---KSLEKEFSEITN------------------GFKEIEKKALYDFKSN-----
B. subtile AlkD2       ----------MNDYTDFPALLEERYSEKIS-------------------FTLIRDDAEEFSRSH-----
B. pyogenes AlkD2      ----------MTIGI---DNILNKIQQIEH------------------GFQHILDGADEIFSTH-----
S. mutans AlkD         --MGCLLCYNQAMDK---KTLIQTFYDHADQERAHAMAAYMRDQFPFLGLSTPLRRQLEKDFVKESKAS
L. sp. oral AlkD       ------------MDF---NKLYEEMIRHKNEEQAQKMSKYMLNKFEYIGIKTPERREIFKIFFKEYKNE
B. pilosicoli AlkD     -------------ML---NDIFEQLTKLQNSKKAKEMSAYMKNKFEFLGVDSKSRKNIENNIFKEYKKT
B. tsurumiense AlkD    ------------MDF---DAVLAMVQAHADERKAQGMRAYMRDQFEFLGVATPVRRASTRPVLREARND
B. coprosuis AlkD      MFYISSPLNINRMNF---NSLLKQYQAVQDKERAAQMEKYMRNQFSFLGIATPERRKIAKPLFQEAKYH
B. cereus AlkD         ----------MHPFV---KALQEHFTAHQNPEKAEPMARYMKNHFLFLGIQTPERRQLLKDIIQIHTLP
                                         Helix A         Helix B                 Helix C
```

Figure S2. Alignment of the ABC and A−C motifs of phylogenetically diverse orthologs of SmAlkD and SmAlkD2.
