# Supplementary material for: A New Family of HEAT-Like Repeat Proteins Lacking a Critical Substrate Recognition Motif Present in Related DNA Glycosylases
Source: PLoS One. 2015 May 15;10(5):e0127733. doi: 10.1371/journal.pone.0127733 (PMC4433238; doi:10.1371/journal.pone.0127733)
Supplement: S3 Fig — (A) Complete protein. (B) A−C motif. The 1.7-Å annealed composite omit map was calculated from the final SmAlkD2 model and contoured at 1σ. (PDF) [file pone.0127733.s003.pdf]

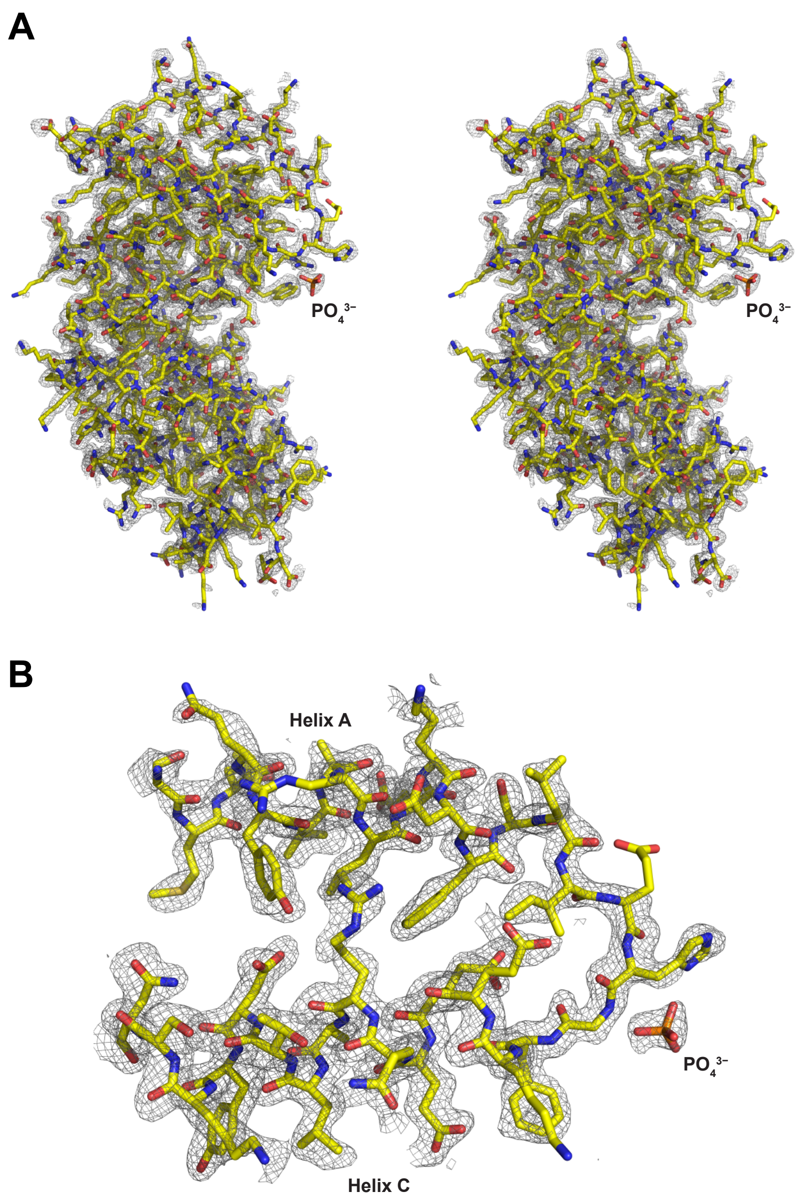


**Figure S3. Electron density for SmAlkD2.** (A) Complete protein. (B) A−C motif. The 1.7-Å annealed composite omit map was calculated from the final SmAlkD2 model and contoured at 1σ.
