# Supplementary material for: A New Family of HEAT-Like Repeat Proteins Lacking a Critical Substrate Recognition Motif Present in Related DNA Glycosylases
Source: PLoS One. 2015 May 15;10(5):e0127733. doi: 10.1371/journal.pone.0127733 (PMC4433238; doi:10.1371/journal.pone.0127733)
Supplement: S4 Fig — (A) B. cereus AlkD. (B) S. mutans AlkD. (C) L. sp. oral AlkD. Homology models in panels B and C were generated from an X-ray crystal structure of BcAlkD (PDB: 3JXY) using SWISS-MODEL [38]. Hydrogen-bonding interactions are indicated with dotted lines. (PDF) [file pone.0127733.s004.pdf]

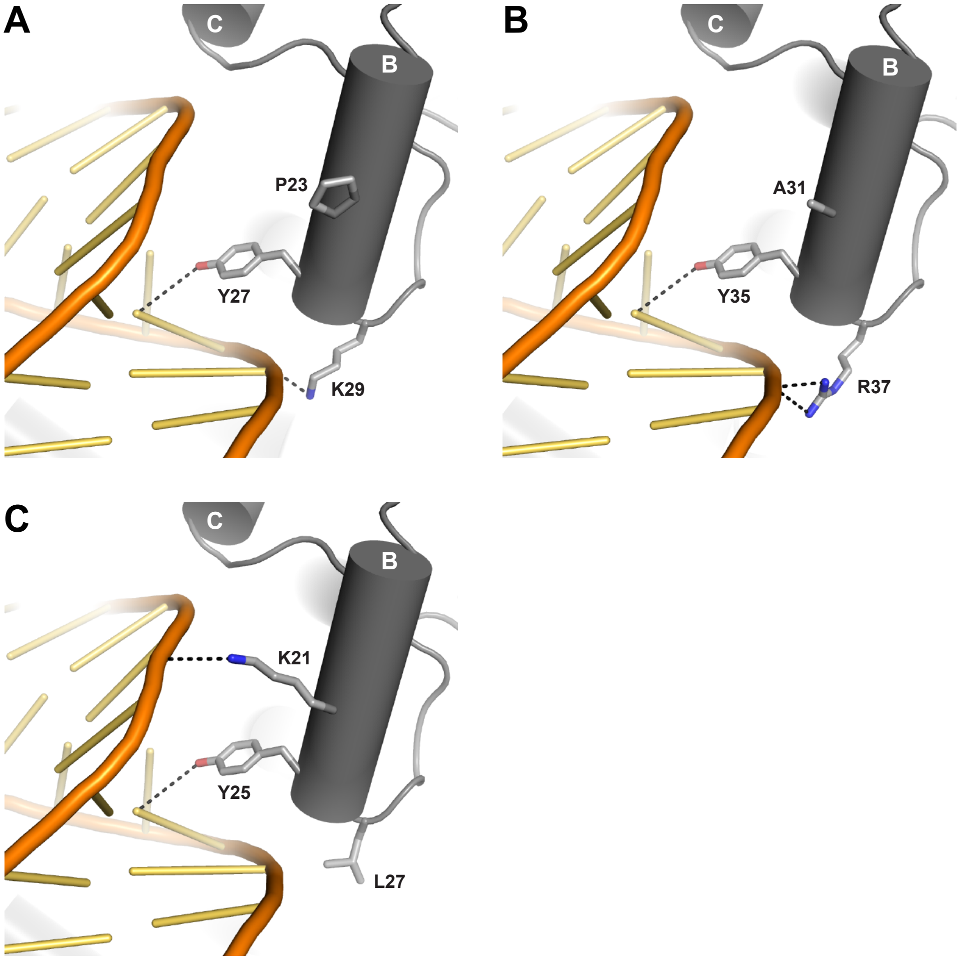

Figure S4. Alternative DNA binding contacts on the B-helix. (A) *B. cereus* AlkD. (B) *S. mutans* AlkD. (C) *L.* sp. oral AlkD. Homology models in panels B and C were generated from an X-ray crystal structure of BcAlkD (PDB: 3JXY) using SWISS-MODEL. Hydrogen-bonding interactions are indicated with dotted lines.
