# Supplementary material for: A New Family of HEAT-Like Repeat Proteins Lacking a Critical Substrate Recognition Motif Present in Related DNA Glycosylases
Source: PLoS One. 2015 May 15;10(5):e0127733. doi: 10.1371/journal.pone.0127733 (PMC4433238; doi:10.1371/journal.pone.0127733)
Supplement: S1 Table — (PDF) [file pone.0127733.s005.pdf]

Table S1. Representative AlkD2 orthologs.

Species	Phylum	E value[a]	Accession no.
Streptococcus mutans	Firmicutes	7×10^{-147}	WP_002267556.1
Leptotrichia sp. oral	Fusobacteria	9×10^{-116}	WP_021766039.1
Brachyspira pilosicoli	Spirochaetes	2×10^{-101}	WP_014932995.1
Bifidobacterium subtile	Actinobacteria	1×10^{-60}	WP_024463156.1
Bacteroides pyogenes	Bacteroidetes	2×10^{-50}	GAE17412.1
Rhodopseudomonas palustris	Alphaproteobacteria	6×10^{-61}	WP_011159812.1
Pasteurella multocida	Gammaproteobacteria	5×10^{-82}	WP_005753048.1
Campylobacter concisus	Epsilonproteobacteria	2×10^{-105}	WP_004317342.1
Synergistes jonesii	Synergistetes	7×10^{-83}	WP_037974974.1
Thermobaculum terrenum	Chloroflexi	4×10^{-50}	WP_012876178.1

[a] Expect (E) values were calculated relative to *Streptococcus mutans* AlkD2.
