# Supplementary material for: A New Family of HEAT-Like Repeat Proteins Lacking a Critical Substrate Recognition Motif Present in Related DNA Glycosylases
Source: PLoS One. 2015 May 15;10(5):e0127733. doi: 10.1371/journal.pone.0127733 (PMC4433238; doi:10.1371/journal.pone.0127733)
Supplement: S2 Table — (PDF) [file pone.0127733.s006.pdf]

**Table S2**. Protein melting temperatures.	
**Protein**	**T_m (°C)**
BcAlkD	40.4[a]
BcAlkD-Y27A	42.6[a]
BcAlkD-K29A	42.1
BcAlkD-Y27A/K29A	45.3
SmAlkD2	55.7

[a] Values were taken from E.A. Mullins, E.H. Rubinson, and B.F. Eichman, The substrate binding interface of alkylpurine DNA glycosylase AlkD, DNA Repair 13 (2014) 50-54.
