# Supplementary material for: A New Family of HEAT-Like Repeat Proteins Lacking a Critical Substrate Recognition Motif Present in Related DNA Glycosylases
Source: PLoS One. 2015 May 15;10(5):e0127733. doi: 10.1371/journal.pone.0127733 (PMC4433238; doi:10.1371/journal.pone.0127733)
Supplement: S3 Table — (PDF) [file pone.0127733.s007.pdf]

Table S3. X-ray data collection and refinement statistics.

**Data collection**	
Space group	$P2_12_12_1$
Cell dimensions	
a, *b*, *c* (Å)	51.02, 65.09, 87.50
α, *β*, *γ* (°)	90.00, 90.00, 90.00
Resolution (Å)	50.00–1.73 (1.76–1.73)[a]
R_{sym}	0.064 (0.383)
Avg. $I/\sigma I$	42.6 (4.8)
Completeness (%)	99.1 (84.1)
Redundancy	8.0 (7.4)
Wilson *B*-factor ($Å^2$)	19.5
**Refinement**	
Resolution (Å)	40.16–1.73 (1.79–1.73)
No. reflections	31,084 (2,738)
R_{work}	0.152 (0.174)
R_{free}[b]	0.168 (0.213)
No. atoms	
Protein	1,735
Buffer[c]	13
Water	271
Avg. *B*-factors[d] ($Å^2$)	
Protein	25.8
Buffer	53.2
Water	41.4
R.m.s. deviations	
Bond lengths (Å)	0.006
Bond angles (°)	0.974
Ramachandran distribution (%)	
Favored	99.1
Allowed	0.9
Disallowed	0.0

[a] Statistics for the highest resolution shell are shown in parentheses.
[b] R_{free} was determined from the 5% of reflections excluded from refinement.
[c] Phosphate, glycerol, sodium, and chloride from the crystallization buffer were included in the model.
[d] Equivalent isotropic *B*-factors were calculated in conjunction with TLS-derived anisotropic *B*-factors.
